# RIPK3-Dependent Recruitment of Low-Inflammatory Myeloid Cells Does Not Protect from Systemic *Salmonella* Infection

**DOI:** 10.1128/mBio.02588-20

**Published:** 2020-10-06

**Authors:** John Satkovich, Christopher J. Anderson, Christopher B. Medina, Matteo Ottolini, John R. Lukens, Melissa M. Kendall

**Affiliations:** a Department of Microbiology, Immunology, and Cancer Biology, University of Virginia School of Medicine, Charlottesville, Virginia, USA; b Department of Pharmacology, University of Virginia School of Medicine, Charlottesville, Virginia, USA; c Department of Neuroscience, Center for Brain Immunology and Glia, University of Virginia School of Medicine, Charlottesville, Virginia, USA; University of Texas Southwestern Medical Center Dallas

**Keywords:** RIPK3, *Salmonella*, bacteria, macrophages, necroptosis, systemic infection

## Abstract

Regulated macrophage death has emerged as an important mechanism to defend against intracellular pathogens. However, the importance and consequences of macrophage death during bacterial infection are poorly resolved. This is especially true for the recently described RIPK3-dependent lytic cell death, termed necroptosis. Salmonella enterica serovar Typhimurium is an intracellular pathogen that precisely regulates virulence expression within macrophages to evade and manipulate immune responses, which is a key factor in its ability to cause severe systemic infections. We combined genetic and pharmacological approaches to examine the importance of RIPK3 for *S.* Typhimurium-induced macrophage death using conditions that recapitulate bacterial gene expression during systemic infection *in vivo*. Our findings indicate that noninvasive *S.* Typhimurium does not naturally induce macrophage necroptosis but does so in the presence of pan-caspase inhibition. Moreover, our data suggest that RIPK3 induction (following caspase inhibition) does not impact host survival following *S.* Typhimurium infection, which differs from previous findings based on inert lipopolysaccharide (LPS) injections. Finally, although necroptosis is typically characterized as highly inflammatory, our data suggest that RIPK3 skews the peritoneal myeloid population away from an inflammatory profile to that of a classically noninflammatory profile. Collectively, these data improve our understanding of *S.* Typhimurium-macrophage interactions, highlight the possibility that purified bacterial components may not accurately recapitulate the complexity of host-pathogen interactions, and reveal a potential and unexpected role for RIPK3 in resolving inflammation.

## Abstract (precis)

Macrophages employ multiple strategies to limit pathogen infection. For example, macrophages may undergo regulated cell death, including RIPK3-dependent necroptosis, as a means of combatting intracellular bacterial pathogens. However, bacteria have evolved mechanisms to evade or exploit immune responses. *Salmonella* is an intracellular pathogen that avoids and manipulates immune detection within macrophages. We examined the contribution of RIPK3 to *Salmonella*-induced macrophage death. Our findings indicate that noninvasive *Salmonella* does not naturally induce necroptosis, but it does so when caspases are inhibited. Moreover, RIPK3 induction (following caspase inhibition) does not impact host survival following *Salmonella* systemic infection. Finally, our data show that RIPK3 induction results in recruitment of low-inflammatory myeloid cells, which was unexpected, as necroptosis is typically described as highly inflammatory. Collectively, these data improve our understanding of pathogen-macrophage interactions, including outcomes of regulated cell death during infection *in vivo*, and reveal a potential new role for RIPK3 in resolving inflammation.

## OBSERVATION

Macrophages are key players in innate immunity that not only ingest and degrade bacterial pathogens but also orchestrate inflammatory responses. Regulated, lytic macrophage death has emerged as an important strategy to resolve infection by eliminating the replicative niche of intracellular pathogens and inducing inflammatory responses important for bacterial clearance (1). Notably, bacterial pathogens have evolved mechanisms to evade and/or exploit immune responses to prolong infection (2) in part through precise and coordinated expression of virulence traits (3).

Salmonella enterica serovar Typhimurium causes acute gastroenteritis that can result in severe systemic infections in immunocompromised hosts (3). *S.* Typhimurium infection can be simply characterized as occurring in two phases: intestinal, in which *S.* Typhimurium invades epithelial cells, and systemic, in which *S.* Typhimurium survives and replicates within macrophages. During infection, *S.* Typhimurium spatiotemporally regulates virulence gene expression during these phases (4) to manipulate host cell processes, including immune responses. Intestinal *S.* Typhimurium expresses a type 3 secretion system (T3SS) and effectors encoded within the *Salmonella* pathogenicity island 1 (SPI-1) (5). SPI-1-expressing *S.* Typhimurium organisms are highly invasive and trigger entry into nonphagocytic epithelial cells. Additionally, SPI-1 expression within epithelial cells induces caspase-1-dependent cell death, called pyroptosis (1, 6–8). Although pyroptosis limits *S.* Typhimurium dissemination (6), concomitant epithelial cell extrusion also provides a means for *S.* Typhimurium to reseed the lumen (7). Conversely, within macrophages, *S.* Typhimurium represses SPI-1 expression (4) and upregulates a second T3SS encoded within SPI-2 to prevent and manipulate immune responses (4), respectively. In agreement with *S.* Typhimurium transcriptional silencing of SPI-1 within macrophages, pyroptosis does not occur within the myeloid cell compartment and does not restrict systemic dissemination during early stages of infection (6).

**FIG 1:**
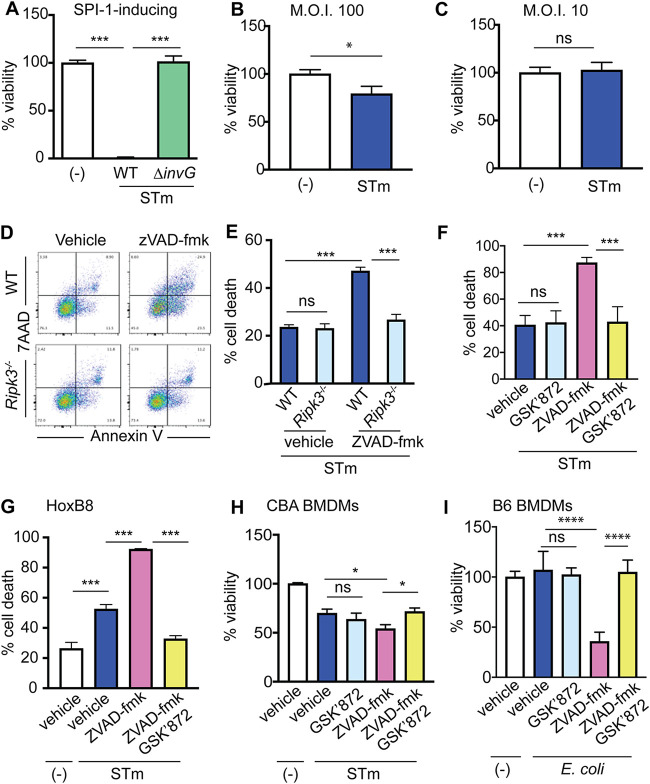
Noninvasive *S.* Typhimurium does not naturally cause RIPK3-dependent death in macrophages. (A) Cell viability of C57BL/6 BMDMs after 2 h mock infection or infection with wild-type (WT) or Δ*invG* (deficient in cell invasion) *S.* Typhimurium strains grown under SPI-1-inducing conditions, as determined by cellular ATP levels. *n* = 15 to 21. (B) Cell viability of C57BL/6 BMDMs after 24 h mock infection or infection with noninvasive *S.* Typhimurium, as determined by cellular ATP levels. *n* = 30. (C) Cell viability of C57BL/6 BMDMs after 24 h mock infection or infection with noninvasive *S.* Typhimurium, as determined by cellular ATP levels. *n* =12. (D) Representative FACS plots showing annexin V (*x* axis) and 7-AAD (*y* axis) staining of noninvasive *S.* Typhimurium-infected WT and *Ripk3*^−/−^ C57BL/6 BMDMs following vehicle or Z-VAD-FMK treatment. (E) Cell viability of vehicle- or Z-VAD-FMK-treated WT and *Ripk3*^−/−^ C57BL/6 BMDMs after 24 h infection with noninvasive *S.* Typhimurium, as determined by flow cytometry. *n* = 6. (F) Cell viability of vehicle-, Z-VAD-FMK-, GSK′872-, or Z-VAD-FMK- and GSK′872-treated C57BL/6 BMDMs after 24 h infection with noninvasive *S.* Typhimurium, as determined by flow cytometry. *n* = 6. (G) Cell viability of vehicle-, Z-VAD-FMK-, GSK′872-, or Z-VAD-FMK- and GSK′872-treated HoxB8 BMDMs after 24 h mock infection or infection with noninvasive *S.* Typhimurium, as determined by flow cytometry. *n* = 3 or 4. (H) Cell viability of vehicle- or GSK′872-treated CBA BMDMs after 24 h mock infection or infection with noninvasive *S.* Typhimurium at a multiplicity of infection (MOI) of 10, as determined by cellular ATP levels. *n* = 9. (I) Cell viability of vehicle- or Z-VAD-FMK-treated C57BL/6 BMDMs after 24 h mock infection or infection with E. coli at an MOI of 10, as determined by cellular ATP levels. *n* = 9. For panels D to F, macrophages were infected with an *S.* Typhimurium at an MOI of 10. For panels A and E to G, statistical significance was determined by ordinary one-way analysis of variance (ANOVA) with Tukey’s multiple-comparison test. For panels B and C, statistical significance was determined by Student's *t* test. For panels H and I, statistical significance was determined by two-way ANOVA with Sidak’s multiple-comparison test. ns, not significant (*P* > 0.05); *, *P* ≤ 0.05; ***, *P* ≤ 0.0005; ****, *P* < 0.0001.

*S.* Typhimurium gene expression can be manipulated *in vitro* using growth conditions that induce or repress SPI-1 expression, resulting in invasive or noninvasive phenotypes, respectively, and this determines the outcome of *S.* Typhimurium-macrophage interactions. Experimental use of invasive *S.* Typhimurium has provided invaluable insights into cellular processes and immune mechanisms, including pyroptosis (9). However, invasive *S.* Typhimurium may not be physiologically relevant (6) and may not reveal processes that influence the outcome of *S.* Typhimurium-macrophage interactions. For example, invasive *S.* Typhimurium causes rapid macrophage death (10) (Fig. 1A), whereas noninvasive *S.* Typhimurium causes macrophage death after 18 to 24 h in a dose-dependent manner (11, 12) (Fig. 1B and C) that was originally described as apoptosis (13). More recently, *S.* Typhimurium was reported to cause necroptosis (14), a programmed form of necrotic cell death that requires the signaling activity of RIPK3, a member of the RIPK family of nonreceptor serine threonine kinases (15). Here, we re-evaluated the importance of RIPK3 for macrophage death during noninvasive *S.* Typhimurium infection and the consequence for infection outcome.

Following infection, noninvasive *S.* Typhimurium caused ∼25% death in both wild-type (WT) and *Ripk3*^−/−^ bone marrow-derived macrophages (BMDMs), based on ATP quantification (Fig. S1A) and lactate dehydrogenase (LDH) release (Fig. S1B). Additionally, *Ripk3*^−/−^ BMDMs displayed levels of annexin V and 7-AAD staining similar to those of WT BMDMs (Fig. 1D and E), indicating that noninvasive *S.* Typhimurium did not trigger RIPK3-dependent necroptosis. Caspase-8 inhibits RIPK3 activity (16) and prevents RIPK3-dependent death in *S.* Typhimurium-infected epithelial cells (17). To test whether caspase inhibition triggers RIPK3-dependent death, BMDMs were harvested from WT and *Ripk3*^−/−^ littermate mice and treated with the pan-caspase inhibitor Z-VAD-FMK. After 18 h of infection with noninvasive *S.* Typhimurium, caspase inhibition resulted in significantly higher levels of death in WT macrophages but not *Ripk3*^−/−^ macrophages (Fig. 1D and E). We confirmed these results by treating WT BMDMs with the pharmacological RIPK3 inhibitor GSK′872. In the absence of caspase inhibition, GSK′872 did not alter *S.* Typhimurium-induced BMDM death compared to vehicle control (Fig. 1F), thus confirming our results obtained from knockout mice. However, GSK′872 prevented BMDM death following caspase inhibition (Fig. 1F). To substantiate these results, we measured cell death of primary macrophages derived from immortalized HoxB8 progenitor cells (18, 19), after *S.* Typhimurium infection and treatment with Z-VAD-FMK alone or in conjunction with GSK′872. These data were similar to results shown in Fig. 1F; however, addition of GSK′872 to Z-VAD-FMK-treated HoxB8 cells resulted in decreased cell death compared to vehicle-treated cells (Fig. 1G). C57BL/6 mice harbor a nonfunctional *nramp-1* gene, which encodes a lysosomal membrane protein in monocytes and macrophages (20) and results in hypersusceptibility to *S.* Typhimurium infection. To examine whether our results depended on Nramp1, we treated BMDMs derived from CBA mice, which carry a functional *nramp-1* gene, with GSK′872 and/or Z-VAD-FMK. These data were similar to data from C57BL/6 macrophages (Fig. 1H) and indicate that in the absence of caspase inhibition, RIPK3 does not influence BMDM viability following *S.* Typhimurium infection regardless of Nramp1. Pan-caspase inhibition (via Z-VAD-FMK) triggers lipopolysaccharide (LPS)-dependent death in macrophages (21). Therefore, to test whether caspase inhibition-induced cell death was specific to *S.* Typhimurium infection, we infected BMDMs with commensal Escherichia coli HS, a strain which was isolated from the stool of a healthy laboratory scientist (22–24). Infection with E. coli in the absence or presence of GSK′872 did not result in macrophage death; however, we measured decreased viability following caspase inhibition, which was rescued by RIPK3 inhibition (Fig. 1I). Altogether, these data indicate that noninvasive *S.* Typhimurium does not naturally cause RIPK3-dependent macrophage death and that caspase inhibition results in RIPK3-dependent death.

10.1128/mBio.02588-20.1 FIG S1 RIPK3 does not influence macrophage death following infection with noninvasive *S.* Typhimurium. (A) Cell viability of *Ripk3*^−/−^ C57BL/6 BMDMs after 24 h mock infection or infection with noninvasive *S.* Typhimurium, as determined by cellular ATP levels. (B) Cell viability of wild-type and *Ripk3*^−/−^ C57BL/6 BMDMs after 24 h infection with noninvasive *S.* Typhimurium, as determined by LDH release. Statistical significance was determined by Student’s t test. ns, not significant (*P* > 0.05); *, *P* ≤ 0.05; **, *P* ≤ 0.005; ***, *P* ≤ 0.0005. Download FIG S1, PDF file, 0.1 MB. Copyright © 2020 Satkovich et al. 2020 Satkovich et al. This content is distributed under the terms of the Creative Commons Attribution 4.0 International license.

**FIG 2:**
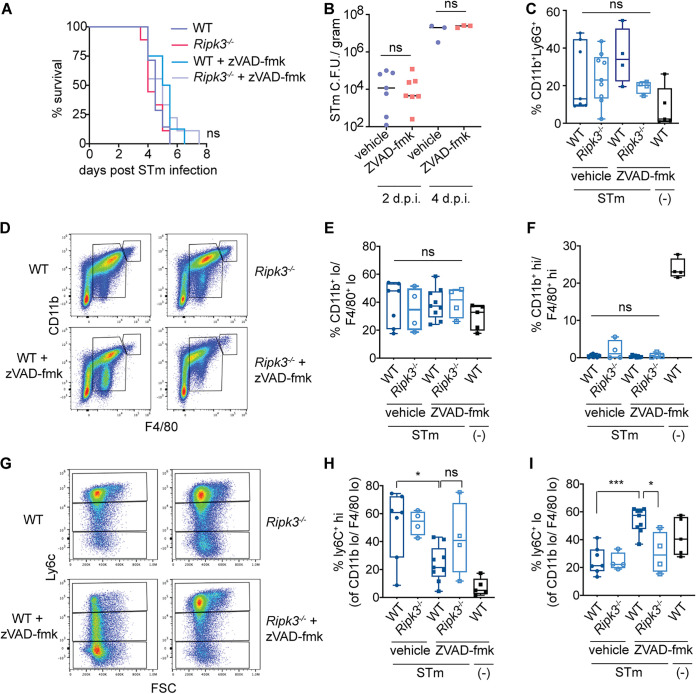
RIPK3 induction during *S.* Typhimurium infection does not affect host survival but results in recruitment of low-inflammatory myeloid cells. (A) Survival curves of vehicle- or Z-VAD-FMK-treated wild-type and *Ripk3*^−/−^ C57BL/6 mice following *S.* Typhimurium infection. *n* = 5 or 6 mice per group/condition. Statistical significance was determined using a log-rank Mantel-Cox test. (B) CFU of *S.* Typhimurium harvested from spleens of vehicle- or Z-VAD-FMK-treated C57BL/6 mice at 2 and 4 dpi. Statistical significance was determined using a Mann-Whitney test. (C) Percentage of neutrophils (CD11b^+^ Ly6G^+^ cells). (D) Representative fluorescence-activated cell sorting (FACS) plots showing CD11b^+^ F4/80^+^ macrophages from *S.* Typhimurium-infected mice. (E) Percentage of CD11b^lo^ F4/80^lo^ macrophages. (F) Percentage of CD11b^hi^ F4/80^hi^ macrophages. (G) Representative FACS plots showing Ly6C expression from *S.* Typhimurium-infected mice. (H and I) Percentage of Ly6C^hi^ (H) and Ly6C^lo^ (I) cells within the CD11b^lo^ F4/80^lo^ macrophage population. For panels C, E, F, H, and I, statistical significance was determined by ordinary one-way ANOVA with Tukey’s multiple-comparison test. ns, not significant (*P* > 0.05); *, *P* ≤ 0.05; ***, *P* ≤ 0.0005.

RIPK3-induced necroptosis has been suggested as a means by which *S.* Typhimurium evades immune responses during systemic infection (14) and thus exacerbates infection. To test this, we intraperitoneally injected *S.* Typhimurium into WT and *Ripk3*^−/−^ littermate mice following vehicle or Z-VAD-FMK treatment (see Fig. S2 for the experimental setup). WT and *Ripk3*^−/−^ mice succumbed to infection at similar rates, and caspase inhibition did not impact outcome (Fig. 2A). In agreement with the *in vitro* macrophage studies, Nramp1 did not affect mouse survival in the context of caspase inhibition (Fig. S3). Consistent with similar mouse survival, *S.* Typhimurium was recovered from the spleens of Z-VAD-FMK- and vehicle-treated mice at similar numbers at 2 and at 4 days postinfection (dpi) (Fig. 2B).

10.1128/mBio.02588-20.2 FIG S2 Schematic of experimental setup for *in vivo* experiments. Download FIG S2, PDF file, 0.2 MB. Copyright © 2020 Satkovich et al. 2020 Satkovich et al. This content is distributed under the terms of the Creative Commons Attribution 4.0 International license.

10.1128/mBio.02588-20.3 FIG S3 Z-VAD-FMK treatment does not affect survival of CBA mice after *S.* Typhimurium infection. Survival curves of vehicle- or Z-VAD-FMK-treated wild-type CBA mice following *S.* Typhimurium infection. *n* = 4. Statistical significance was determined using a log-rank Mantel-Cox test. Download FIG S3, PDF file, 0.04 MB. Copyright © 2020 Satkovich et al. 2020 Satkovich et al. This content is distributed under the terms of the Creative Commons Attribution 4.0 International license.

The manner in which a cell dies can influence subsequent immune signaling and inflammation (1). Profiling of the immune cells (based on the gating strategy shown in Fig. S4) in the peritoneal cavity at 2 dpi indicated similar numbers of neutrophils as well as large and small peritoneal macrophages in Z-VAD-FMK-treated infected mice compared to vehicle-treated infected mice (Fig. 2C to F). Next, we further sorted the monocytes based on Ly6C expression. Ly6C^hi^ (Ly6C^hi^ CCR2^hi^ CX_3_CR1^lo^) monocytes are often referred to as inflammatory monocytes, in contrast to Ly6C^lo^ (Ly6C^lo^ CCR2^lo^ CX_3_CR1^hi^) monocytes (25). Ly6C^hi^ monocytes directly contribute to host defense via the production of antimicrobial inducible nitric oxide synthase (iNOS) and indirectly through the expression of proinflammatory cytokines and stimulation of the adaptive immune response (25). In contrast, less is known about the role of Ly6C^lo^ monocytes during bacterial infection. In WT mice, Ly6C^hi^ monocytes were recruited during *S.* Typhimurium systemic infection (26, 27) (Fig. 2G and H). Notably, we measured an increase in Ly6C^lo^ cells and a corresponding decrease in the proportion of Ly6C^hi^ cells in Z-VAD-FMK-treated wild-type mice compared to vehicle-treated, *S.* Typhimurium-infected mice (Fig. 2G to I). Caspase inhibition-induced Ly6C^lo^ myeloid population dynamics were dependent on RIPK3, as no differences in Ly6C populations were measured in Z-VAD-FMK-treated *Ripk3*^−/−^ mice compared to infection-matched, vehicle-treated *Ripk3*^−/−^ mice (Fig. 2G to I). Shifts in myeloid populations have recently been observed in mice given systemic LPS; however, the importance of RIPK3 for this finding was not tested (28). Moreover, although caspase inhibition protected mice from inert LPS (28), our studies suggest that RIPK3 induction does not protect mice from active Gram-negative bacterial infection or limit bacterial burden.

10.1128/mBio.02588-20.4 FIG S4 Flow cytometry gating strategy for the peritoneal lavage from *S.* Typhimurium or mock-infected wild-type and *Ripk3*^−/−^ mice at 2 dpi with Z-VAD-FMK or vehicle treatments every 12 h. Total peritoneal lavage was gated on the cellular population (FSC-A/SSC-A) and singlets (FSC-A/FSA-H) to exclude cell debris and doublets. Single cells were then gated on 7-AAD to obtain the 7-AAD-negative live cell population. CD11b^+^ Ly6G^+^ was used to identify neutrophils, and the Ly6G^−^ population was further analyzed for macrophage markers. The F4/80^hi^ CD11b^hi^ population was used to identify peritoneal macrophages. The CD11b^lo^ F4/80^lo^ myeloid cells population was further analyzed based on Ly6C expression. Download FIG S4, PDF file, 0.6 MB. Copyright © 2020 Satkovich et al. 2020 Satkovich et al. This content is distributed under the terms of the Creative Commons Attribution 4.0 International license.

Collectively, our data indicate that noninvasive *S.* Typhimurium does not trigger RIPK3-dependent macrophage death and that when induced, RIPK3 does not influence systemic *S.* Typhimurium infection. These data highlight the ability of *S.* Typhimurium to evade immune detection to maintain its reservoir. Here, we accounted for littermate controls, different vivaria (University of Virginia and VIB/University of Gent), and mouse genetic backgrounds. Furthermore, our study reveals that necroptosis (resulting from the use of a pan-caspase inhibitor) leads to differential myeloid populations during infection but fails to protect the host from the outcomes of acute infection. Ly6C^lo^ cells are associated with anti-inflammatory processes (29), including production of the anti-inflammatory cytokine interleukin 10 (IL-10) (30), which suggests that RIPK3 activation may contribute to the resolution of low-grade or chronic infection. This idea is consistent with a recent study that reported increased levels of IL-10 production by macrophages deficient in the pyroptotic executioner caspases 1 and 11 *in vitro* (31). Altogether, these data underscore a role for necroptosis in limiting excessive inflammation during bacterial infection (32); however, this idea requires additional investigation.

Materials and methods are provided in Text S1.

10.1128/mBio.02588-20.5 TEXT S1 Supplemental materials and methods. Download Text S1, PDF file, 0.1 MB. Copyright © 2020 Satkovich et al. 2020 Satkovich et al. This content is distributed under the terms of the Creative Commons Attribution 4.0 International license.
