# Supplementary material for: RIPK3-Dependent Recruitment of Low-Inflammatory Myeloid Cells Does Not Protect from Systemic Salmonella Infection
Source: mBio. 2020 Oct 6;11(5):e02588-20. doi: 10.1128/mBio.02588-20 (PMC7542371; doi:10.1128/mBio.02588-20)
Supplement: FIG S1 [file mBio.02588-20-sf001.pdf]

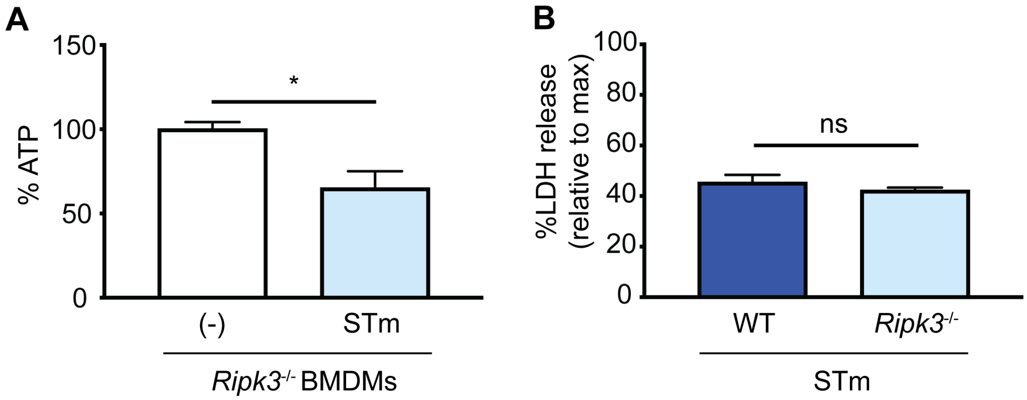

Fig. S1. RIPK3 does not influence macrophage death following infection with noninvasive STm. A) Cell viability of *Ripk3*$^{-/-}$ C57BL/6 BMDMs after 24 h mock-infection or infection with noninvasive STm as determined by cellular ATP levels. B) Cell viability of wildtype and *Ripk3*$^{-/-}$ C57BL/6 BMDMs after 24 h infection with noninvasive STm as determined by LDH release. Statistical significance was determined by Student's t test. For all statistical significance, ns = $p > 0.05$, * $p \leq 0.05$, ** $p \leq 0.005$, *** $p \leq 0.0005$.
