# Supplementary figures and images for: RIPK3-Dependent Recruitment of Low-Inflammatory Myeloid Cells Does Not Protect from Systemic Salmonella Infection
Source: mBio. 2020 Oct 6;11(5):e02588-20. doi: 10.1128/mBio.02588-20 (PMC7542371; doi:10.1128/mBio.02588-20)

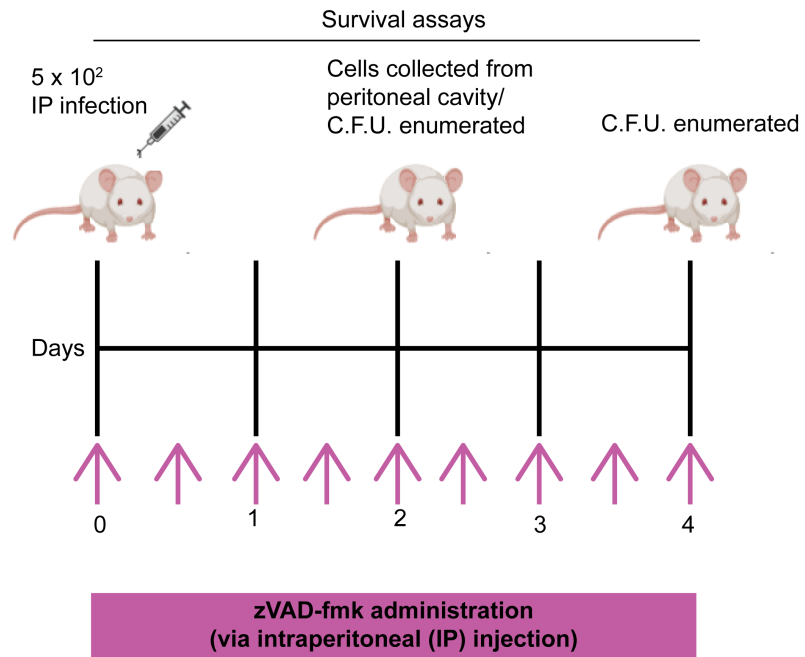

**Fig. S2.** Schematic of experimental setup for *in vivo* experiments.

Supplement: FIG S2 [file mBio.02588-20-sf002.pdf]
