# Supplementary material for: RIPK3-Dependent Recruitment of Low-Inflammatory Myeloid Cells Does Not Protect from Systemic Salmonella Infection
Source: mBio. 2020 Oct 6;11(5):e02588-20. doi: 10.1128/mBio.02588-20 (PMC7542371; doi:10.1128/mBio.02588-20)
Supplement: FIG S3 [file mBio.02588-20-sf003.pdf]

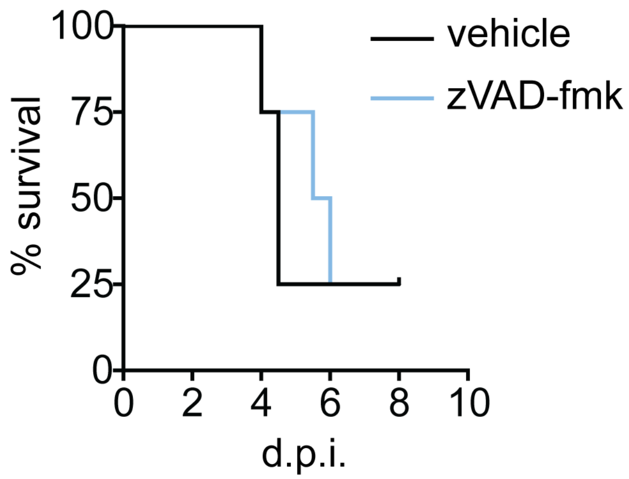

Fig. S3. zVAD-fmk treatment does not affect survival of CBA mice after STm infection. Survival curves of vehicle- or zVAD-fmk-treated wildtype CBA mice following STm infection. n=4. Statistical significance was determined using a long-rank Mantel-Cox test.
