# Supplementary material for: RIPK3-Dependent Recruitment of Low-Inflammatory Myeloid Cells Does Not Protect from Systemic Salmonella Infection
Source: mBio. 2020 Oct 6;11(5):e02588-20. doi: 10.1128/mBio.02588-20 (PMC7542371; doi:10.1128/mBio.02588-20)
Supplement: FIG S4 [file mBio.02588-20-sf004.pdf]

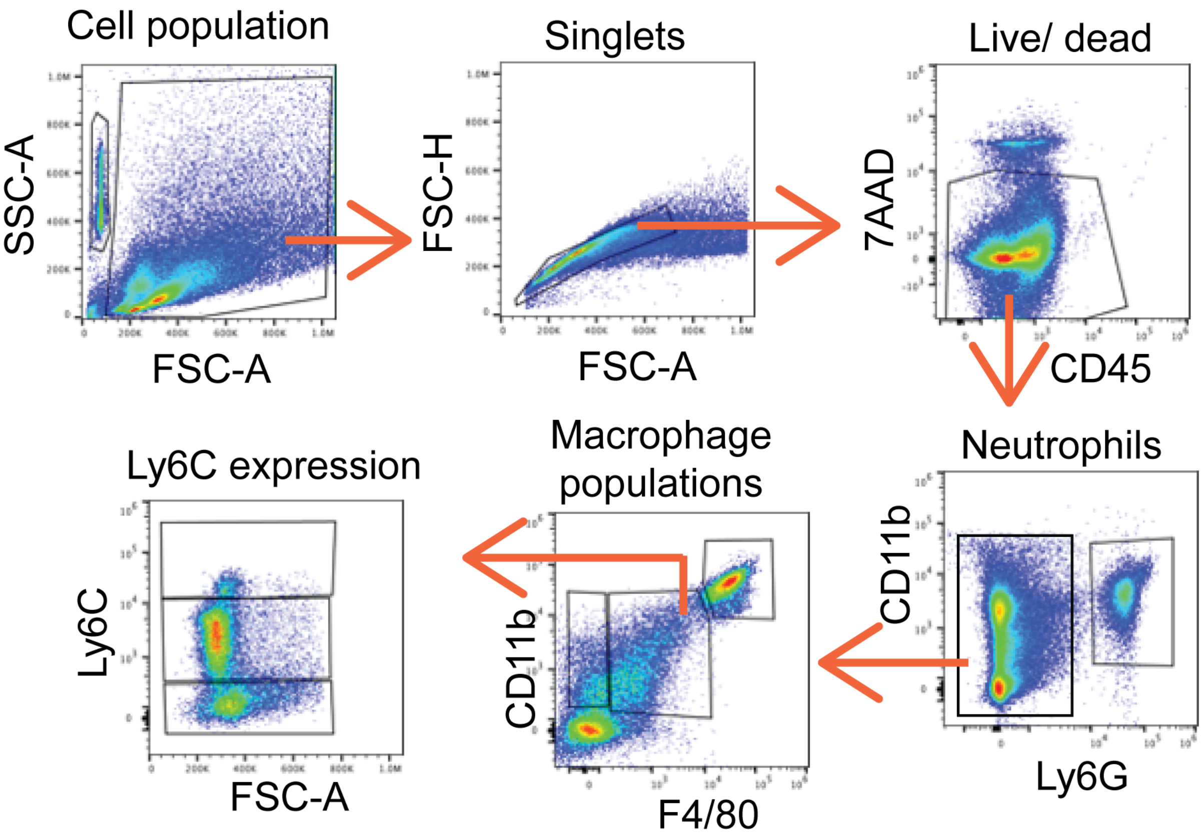

Fig. S4. Flow Cytometry gating strategy for the peritoneal lavage from STm or mock-infected wildtype and *Ripk3*$^{-/-}$ mice at 2 d.p.i. with zVAD-FMK or vehicle treatments every 12 hours. Total peritoneal lavage was gated on the cellular population (FSC-A/SSC-A) and singlets (FSC-A/FSA-H) to exclude cell debris and doublets. Single cells were then gated on 7AAD to obtain the 7AAD negative live cell population. CD11b+LyG+ was used to identify neutrophils and the Ly6G- population was further analyzed for macrophage markers. F4/80hiCD11bhi population was used to identify peritoneal macrophages. The CD11bloF4/80lo myeloid cells population was further analyzed based on Ly6C expression.
