# Supplementary material for: RIPK3-Dependent Recruitment of Low-Inflammatory Myeloid Cells Does Not Protect from Systemic Salmonella Infection
Source: mBio. 2020 Oct 6;11(5):e02588-20. doi: 10.1128/mBio.02588-20 (PMC7542371; doi:10.1128/mBio.02588-20)
Supplement: TEXT S1 [file mBio.02588-20-s0001.pdf]

MATERIALS AND METHODS

Bacterial culture

Salmonella enterica serovar Typhimurium (STm) stain SL1344 (1), the isogenic ∆*invG* STm strain (2), and wildtype *Escherichia coli* strain HS (3) were used in this study. Bacterial cultures were routinely grown in LB broth overnight. Following overnight growth, cultures were washed and resuspended in 1x PBS prior to use. With the exception of data shown in Figure 1A, STm was not grown under SPI-1 inducing conditions *in vitro* prior to mouse or macrophage infection in order to mimic STm gene expression during the systemic phase of infection (4-7).

Mammalian cell culture conditions

Bone marrow-derived macrophages (BMDMs) of indicated genotype were extracted and differentiated as previously described (4). Bone marrow was flushed from the hind limbs of 8-10 week old mice of the indicated genotype. Bone marrow progenitor cells were incubated in RPMI supplemented with 10% heat-inactivated FBS, 1x penicillin-streptomycin-glutamine, and 20% L929 conditioned media (as a source of colony-stimulating factor 1). Fresh media was provided on day 3 of differentiation before cells were washed and seeded for use after day 6 of differentiation. L929 conditioned media and antibiotics were omitted from the media for all experiments following differentiation. HoxB8 progenitor cells were derived as previously described (8, 9). For subsequent differentiation and use, HoxB8 progenitors were cultured in RPMI supplemented with 10% heat-inactivated FBS, 1x penicillin-streptomycin-glutamine, and 50ng/ml of M-CSF for 6 days, with fresh media provided on day 3. Purified M-CSF was provided by the VIB Protein Core

(VIB-UGent Inflammation Research Center). Purified M-CSF and antibiotics were omitted from the media for all experiments following differentiation. BMDMs and HoxB8 macrophages were seeded at densities of 4e4 cells/well (96-well plates), 2e5 cells/well (24-well plates), or 5e5 cells/well (12-well plates).

For Caspase inhibition, macrophages were pretreated with 10mM of the pan-Caspase inhibitor zVAD-FMK (Enzo Life Sciences) or vehicle control (DMSO) for 1 h. zVAD-FMK was omitted from the media during the initial 1 h of bacterial phagocytosis (see below) but was provided for the remainder of the experiment. For RIPK3 inhibition, macrophages were pretreated with 5μM of GSK'872 (Selleckchem) or vehicle control (DMSO) for 1 h. GSK'872 was omitted from the media during the initial 1 h of bacterial phagocytosis (see below) but was provided for the remainder of the experiment.

Macrophage viability assays

Flow cytometry. Infection-induced macrophage cell death was quantified following a standard gentamicin protection assay (10). Macrophages were infected with a multiplicity of infection (M.O.I.) of 10 STm (ratio of bacterial organism to mammalian macrophage) as listed and spun down at 1000 rpm for 1 min to increase phagocytosis. Following 1 h incubation, gentamicin-containing media (100μg/ml) was provided for 30 min to kill any extracellular bacteria. A final culture media containing 10μg/ml of gentamicin was provided for the duration of the experiment. At the indicated time, BMDM supernatant (to recover any floating cells) and cells were harvested. Samples were spun down and the cell pellet was resuspended in Annexin V binding buffer containing Annexin V-Pacific blue or Annexin V-APC (Biolegend) and 7AAD (Thermo Fischer) to stain for cell death. After staining cells were washed

with Annexin V binding buffer and immediately placed on ice. Sample acquisition was performed on the BD FACSCanto I (BD Biosciences) and FlowJo v. 10.4 was used for analysis.

ATP production. Macrophages were infected as described above. At the indicated time post-infection, media supernatant was removed and cells were washed once with 1x PBS. Macrophage ATP production was measured via the CellTiter-Glo® Luminescent Cell Viability Assay (Promega) according to the manufacturer’s instructions. % ATP was calculated and normalized such that uninfected and untreated macrophages were set to 100%.

LDH release. Macrophages were infected as described above. At the indicated time post-infection, media supernatant was removed and LDH was detected using the CytoTox 96® Non-Radioactive Cytotoxicity Assay (Promega) according to the manufacturer’s instructions. Maximum LDH release was included using the provided lysis solution and experimental data were calculated and reported as a percent of the maximum release control.

Animal studies

All animal experiments were approved by Institutional Animal Care and Use Committee at the University of Virginia School of Medicine. WT C57BL/6NJ and WT CBA/J mice were purchased from Jackson Laboratories and Envigo. *Ripk3*$^{-/-}$ and corresponding WT littermate control animals were described previously (11) and maintained at the University of Virginia. Mice received an intraperitoneal (IP) injection of 0.1mg zVAD-FMK or vehicle control (DMSO) every 12 h starting at the time of bacterial infection for the duration of the experiment.

Animal survival. 8-10 Week old WT and *Ripk3*$^{-/-}$ littermate C57BL/6NJ or CBA mice were infected via ip injection with 5e2 colony forming units (CFU) of STm (as described above). Mouse body weight and morbidity was measured every 12 h. Mice were sacrificed once they lost greater than 20% of their initial starting body weight, or if morbidity exceeded published criteria for humane euthanasia (12).

Bacterial burden in vivo. 8-10 week old female C57BL/6NJ mice were infected via ip injection with 5e2 CFU of STm (as described above). Mice were euthanized and organs were harvested on day 2 post-infection. Spleen and liver were harvested and homogenized in sterile 1x PBS. Serial dilutions were plated onto MacConkey agar containing streptomycin for CFU enumeration.

Immunophenotyping in vivo. For peritoneal lavage, 10mls of PBS (5%BSA) was injected in the peritoneum then extracted using a fresh needle. Total extraction volume was measured. RBC lysis was performed where required. Samples were spun down and resuspended in FACS Buffer (PBS 1% BSA) with FcBlock (CD16/CD32) (Ebiosceince) for 20 min on ice to inhibit non-specific antibody binding. After FcBlock samples were resuspended in FACS buffer with; Ly-6C-eFluor-450 (Ebiosceince), Siglec-F-PE (BD), Ly-6G-APC (Ebiosceince), F4/80-FITC (Ebiosceince), CD11b-APC-Cy7 (BD), and CD45-PerCP (BD) to determine immune cell populations. Sample acquisition was performed on the Attune Nxt cytometer (Thermo Fischer) and FlowJo v. 10.4 was used for analysis.

REFERENCES

1. Hoiseth SK, Stocker BA. 1981. Aromatic-dependent *Salmonella typhimurium* are non-virulenct and effective as live vaccines. Nature 291:238-239.
2. Criss AK, Ahlgren DM, Jou TS, McCormick BA, Casanova JE. 2001. The GTPase Rac1 selectively regulates *Salmonella* invasion at the apical plasma membrane of polarized epithelial cells. J Cell Sci 114:1331-1341.
3. Levine MM, Bergquist EJ, Nalin DR, Waterman DH, Hornick RB, Young CR, Sotman S, Rowe B. 1978. *Escherichia coli* strains that cause diarrhoea but do not produce heat-labile or teat-stable enterotoxins and are non-invasive. Lancet i:1119-1122.
4. Anderson CJ, Satkovich J, Köseoğlu V, Agaisse H, Kendall MM. 2018. The Ethanolamine Permease EutH Promotes Vacuole Adaptation of *Salmonella enterica* and *Listeria monocytogenes* during Macrophage Infection. Infect Immun 86:pii: e00172-18.
5. Hausmann A, Böck D, Geiser P, Berthold DL, Fattinger SA, Furter M, Bouman JA, Barthel-Scherrer M, Lang CM, Bakkeren E, Kolinko I, Diard M, Bumann D, Slack E, Regoes RR, Pilhofer M, Sellin ME, Hardt WD. 2020. Intestinal epithelial NAIP/NLRC4 restricts systemic dissemination of the adapted pathogen Salmonella Typhimurium due to site-specific bacterial PAMP expression. Mucosal Immunol doi:doi: 10.1038/s41385-019-0247-0.
6. Laughlin RC, Knodler LA, Barhoumi R, Payne HR, Wu J, Gomez G, Pugh R, Lawhon S, Baumler AJ, Steele-Mortimer O, Adams LG. 2014. Spatial segregation of virulence gene expression during acute enteric infection with *Salmonella enterica* serovar Typhimurium. mBio 5:e00946-13.

7. Owen K, Meyer CB, Bouton AH, Casanova JE. 2014. Activation of focal adhesion kinase by *Salmonella* suppresses autophagy via an Akt/mTOR signaling pathway and promotes bacterial survival in macrophages. PLoS Pathog 10:e1004159.
8. Wang GG, Calvo KR, Pasillas MP, Sykes DB, Häcker H, Kamps MP. 2006. Quantitative production of macrophages or neutrophils *ex vivo* using conditional Hoxb8. Nat Methods 3:287-293.
9. Redecke V, Wu R, Zhou J, Finkelstein D, Chaturvedi V, High AA, Hacker H. 2013. Hematopoietic progenitor cell lines with myeloid and lymphoid potential. Nat Methods 10:795-803.
10. Anderson CJ, Clark DE, Adli M, Kendall MM. 2015. Ethanolamine signaling promotes *Salmonella* niche recognition and adaptation during infection. PLoS Pathog 11:e1005278.
11. Newton K, Sun X, Dixit VM. 2004. Kinase RIP3 is dispensable for normal NF-κBs, signaling by the B-cell and T-cell receptors, tumor necrosis factor receptor 1, and Toll-like receptors 2 and 4. Mol Cell Biol 24:1464–1469
12. Owen KA, Anderson CJ, Casanova JE. 2016. *Salmonella* Suppresses the TRIF-Dependent Type I Interferon Response in Macrophages. MBio 7:e02051-15.
